# Mutations in the BRAF, NRAS, and C-KIT Genes of Patients Diagnosed with Melanoma in Colombia Population

**DOI:** 10.1155/2020/2046947

**Published:** 2020-07-22

**Authors:** Luz D. Gutiérrez-Castañeda, Mauricio Gamboa, John A. Nova, Leonardo Pulido, Jose D. Tovar-Parra

**Affiliations:** Hospital Universitario-Centro Dermatológico Federico Lleras Acosta-CDFLLA, Bogota 111511, Colombia

## Abstract

**Introduction:**

Mutations in the BRAF, NRAS, and C-KIT genes have been associated with the histopathological characteristics of melanoma. Likewise, the incidence of each of these subtypes changes according to the geographical origin of the population analyzed.

**Objective:**

To determine the mutation frequency in exons 11 and 15 of the BRAF gene, exons 1 and 2 of the NRAS gene, and exons 11, 13, and 17 of the C-KIT gene and to relate it with histological subtypes in patients from a region with high levels of ultraviolet radiation. *Methodology*. The clinicopathological characteristics of 54 cutaneous melanoma samples were analyzed. Mutation analysis was performed via qPCR on paraffin-embedded tumor tissue samples using probes specific for the V600E mutation. Amplification of exons 11 and 15 of the BRAF gene, exons 1 and 2 of the NRAS gene, and exons 11, 13, and 17 of the C-KIT gene was performed for subsequent sequencing using the Sanger method.

**Result:**

The most frequent histological subtype in the analyzed sample was lentigo maligna/lentigo maligna melanoma (52%) followed by acral lentiginous melanoma (20%). The BRAF-V600 variant was the most frequent (63.6%). The most frequent (54%) mutation in NRAS was p.Lys5∗. In the C-KIT gene, only the Val560Ala mutation was found.

**Conclusion:**

Differences in histological subtypes and mutations in the BRAF, NRAS, and C-KIT genes were found in the analyzed population. This indicates that environmental and genetic factors significantly influence the introduction of the disease in this geographic region.

## 1. Introduction

In recent decades, an increase in the incidence of cutaneous melanoma has been reported [1]. Incidence rates are approximately 60% higher in men than in women [2] and, in particular, have increased in younger age groups (<50 years) [1]. There are differences in the incidence and anatomical distribution of melanoma according to race/ethnicity. This cancer occurs more commonly in Caucasian individuals than in people of other races/ethnicities; however, non-Caucasian individuals tend to present a more advanced stage at the time of diagnosis and have a lower 5-year survival rate than do Caucasians [3]. According to the data from Globocan, in 2018, 1907 new melanoma cases and 518 deaths were reported. Melanoma is the 14th most frequent malignant tumor in Colombia [4]. In South America, the incidence and mortality are estimated of 0.29% and 0.8%, respectively. This is less than in North America (1.4% and 0.15%) but higher than in Central America (0.19% and 0.05%) [5].

Melanoma develops due to a complex interaction between genetic and environmental risk factors, which not only determine melanoma incidence but also influence clinical characteristics and oncogenic pathways. For example, although one of the external factors associated with the development of this disease is ultraviolet radiation (UVR), melanoma incidence is inversely proportional to distance from the equator [3]. Colombia is a country located in South America, in the equatorial line where the melanoma incidence is low compared to that reported worldwide [6]. In addition, the histological subtypes of melanoma with higher frequency in this geographic region are different from those reported in regions with high melanoma frequencies [6]. This indicates that the risk factors associated with melanoma have different roles according to the ethnic origin of the sample analyzed (or with the geographic region analyzed).

Prolonged activation of the RAS/RAF/MAPK pathway and the PI3K pathway plays an important role in the key cellular signaling mechanisms for the transformation of melanocytes; these mutations induce overactivation of the intracellular network of downstream signaling cascades to promote the abnormal growth of melanocytes [7]. Different studies have linked mutually exclusive mutations in the *BRAF*, *NRAS*, *C-KIT*, and *NF-1* genes with the development of melanoma [8, 9]. According to a meta-analysis conducted in 2010 by Lee et al. [10], the mutation frequency in the *BRAF* gene was 41% and that in the *NRAS* gene was 18%. Mutations in the *C-KIT* gene occur in 28 and 36% of mucosal melanoma and acral lentiginous melanoma cases [11, 12]. The most common mutation in the *BRAF* gene is V600E (T1799A; substitution of valine for glutamic acid), with a frequency between 60 and 80%, followed by V600K and V600R, with approximate frequencies of 20% [13, 14]. Non-V600 *BRAF* mutations occur in 3-5% of melanomas [15]. The V600K mutation is found more frequently in patients over 65 years of age, is related to exposure to UVR, and contributes to demographic variations in prevalence [16]. In the *NRAS* gene, the most common mutations are p.G12D and p.Q61K in exons 1 and 2, respectively, with a frequency between 20 and 30% [17, 18]. Mutations in different exons of the *C-KIT* gene occur in approximately 15% of malignant melanomas (MM) cases [19]. According to these mutations and with the data published by The Cancer Genome Atlas (TCGA) in a complete exome sequence analysis of samples from 333 individuals with primary and metastatic melanoma, melanomas can be classified into 4 genomic groups: *BRAF* mutants, *NRAS* mutants, *NF1* mutants (gene for neurofibromatosis type 1), and triple wild-type mutants. These subtypes may be of predictive value given the availability of target therapy for tumors with these molecular alterations [20].

According to the above, genetic alterations constitute one of the main risk factors for the development of melanoma. In Colombia, it is unknown whether alterations in these genes are directly related to the increase in melanoma incidence in the Colombian population. To date, only one study of Colombian patients is known, in which samples of uveal melanoma and primary and metastatic cutaneous melanomas were genotyped, finding results different from those reported in the worldwide population [21]. Therefore, the objective of this study is to describe the demographic and clinicopathological characteristics and to analyze the mutations in exons 11 and 15 of the *BRAF* gene, exons 1 and 2 of the *NRAS* gene, and exons 11, 13, and 17 of the *C-KIT* gene in samples of patients diagnosed with cutaneous melanoma.

## 2. Materials and Methods

### 2.1. Cell Lines

The HT-29 (ATCC® HTB-38), A375, THP-1, SK-SH5Y, and HEP2 cell lines were used as controls for the analyzed variants. All cell lines were cultured in RPMI medium (Gibco ®) with 10% FBS at 37°C and 5% CO2.

### 2.2. Cutaneous Melanoma Samples from Patients

This study was approved by the research ethics committee of the University Hospital-CDFLLA and was conducted in accordance with national and international ethical standards. Each patient signed an informed consent form for the use of their clinical and pathology information and for the analysis of mutations. Histological and clinical data were retrieved retrospectively and prospectively from clinical records. Patients were invited to participate in the study, and a survey was conducted to acquire patient lifestyle and disease progression data. The data were recorded in a database and included demographic records, tumor site, histological type, Breslow's depth, Clark level, and sun exposure. A total of 54 paraffin-embedded tissue samples from the tumor biopsies of each patient, which were obtained from the pathology sample bank of the University Hospital-CDFLLA, were analyzed. Samples collected from 2012 to 2018 were included. A histological section was made for each sample, and the diagnosis was confirmed by a different pathologist than the one who made the initial diagnosis.

### 2.3. DNA Extraction

DNA was isolated from 5 *μ*m thick sections of paraffin-embedded tissue samples. A pathologist demarcated the area from which microdissection was performed, ensuring more than 80% tumor tissue. Subsequently, the samples were deparaffinized in xylol. For DNA extraction, the QIAamp® DNA FFPE Tissue Handbook was used according to the manufacturer's protocol.

### 2.4. Mutation Analysis

Detection of V600 mutations in the BRAF gene and p.G12D, p.G12T, and p.G13D mutations in the NRAS gene.

Recurrent mutations were identified by qPCR using specific hybridization probes to detect the following variants: V600E in the BRAF gene and p.Q61K (codon 61; exon 2) and p.G12D, p.G12T, and p.G13D (codons 12 and 13; exon 1) in the NRAS gene. As a positive control, cell lines containing these variants were used. The HT29 and A375 cell lines carry the p.V600E mutation; SH-SY5Y cells carry mutation p.Q61K; THP-1 cells carry the p.G12D mutation; and Hep-2 cells express the wild-type versions of the mutations analyzed. The detection of the variants was performed using a LightCycler® 96 real-time PCR machine. The probe LightCycler® FastStart DNA Master Hybprobe (ROCHE®) was used. The following primers were used: BRAF V600E, F-5′-CTCTTCATAATGCTTGCTCTGATAGG-3′ and R-5′TAGTAACTCAGCAGCATCTCAGG-3′; NRAS p.Q61K, F-5′GCTGGTGTGAAATGACTGAG-3′ and R-5′-GATGATCCGACAAGTGAGAG-3′; and NRAS p.G12T and p.G13D, F-5′-CCTGTTTGTTGGACATACTG-3′ and R-5′-CCTGTAGAGGTTAATATCCG-3′.

### 2.5. Conventional PCR and Sequencing

To detect mutations different from the recurrent mutations in exons 1 and 2 of the NRAS gene, exon 15 of the BRAF gene, and exons 11, 13, and 17 of the C-KIT gene, segments including the entire exon were amplified and subsequently sequenced by the Sanger method. The amplification of genomic DNA was performed by PCR; the primers were designed using Primers3 software. The following primers were used: exon 11 of the C-KIT gene, F-5′-CCAGAGTGCTCTAATGACTG-3′and R-5′-ACCCAAAAAGGTGACATGGA-3′; exon 13-C-KIT F5′-GCGTAAGTTCCTGTATGGTA-3′and R-5′-AACCTGACAGACAATAAAAG-3′; exon 17-C-KIT F-5′-TGATTTTTATTTTTGGTGTACTGA-3′and R-5′-ACTGTCAAGCAGAGAGAATGGGT-3′; exon 11 of the BRAF gene, F-5′-CTCTTCATAATGCTTGCTCTGATAGG-3′, and R-5′-TAGTAACTCAGCAGCATCTCAGG-3′; and exon 15 of the BRAF gene, F-5′-CTCTTCATAATGCTTGCTCTGATAGG-3′, and R-5′-TAGTAACTCAGCAGCATCTCAGG-3. The PCR contained 2.5 mM dNTPs, 5 U/ml TopTaq DNA polymerase (Qiagen®), 50 pmol/ml each primer, 10X buffer, 25 mM MgCl2, and 400 ng of DNA. The PCR conditions were as follows: initial denaturation for 3 min at 95°C; 40 cycles of denaturation for 30 sec at 95°C, hybridization for 30 sec (between 53°C and 65°C depending on the exon), and 30 sec extension at 72°C; and a final extension for 3 min at 72°C. The PCR products were purified using a QIAquick PCR purification kit (Qiagen®). Sequencing was performed with the Sanger method using a BigDye Terminator V1.1 Cycle Sequencing Kit and an ABI PRISM 3130xl Genetic Analyzer (Applied Biosystems ®). Sequence analysis was performed using the software Chromas (free version) and NovoSNP version 3.0 (free version from the internet, http://www.molgen.ua.ac.be/bioinfo/novosnp/index.html). All sequences were confirmed by a second reviewer.

## 3. Results

### 3.1. Analysis of the Clinical-Demographic Data and Histological Data

Of the 54 patients included in the study, 63% (34/54) were women, and 37% (20/54) were men; the mean age was 62 years (27-86 years). Fifty percent of the patients were between 41 and 79 years old.

The most frequent histological subtype was lentigo maligna/lentigo maligna melanoma (LM/LMM), with 52% (28/54), followed by acral lentiginous melanoma (ALM), with 24% (13/54), nodular melanoma (NM), with 18.5% (10/54), and superficial spreading melanoma (SSM), with 5.5% (3/54). From histologic subtype, the percentage of women for each diagnostic was 61% (17/28) for LM/LMM, 62% (8/13) for ALM, 60% (6/10) for NM, and 100% (3/3) for SSM.

With respect to phototypes, the most frequent phototype was phototype 2, with 50% (27/54), followed by phototype 3, with 36%. In total, 46% (13/28) and 42% (12/28) of patients with the LLM subtype had phototypes 2 and 3, respectively. For ALM, the most frequent phototype was 3, in 46% (6/13) of cases, followed by 2, in 30% (4/13) of the cases. For nodular melanoma, 8 of 10 patients (80%) had phototype 2, and 1 patient had phototype 3. The most frequent location was in the head and neck, 48% (26/54), followed by hands and feet, 30% (13/54), and the trunk, 15% (8/54). The frequency of occurrence in the extremities was 7%.

Four patients had a history of cancer: one had basal cell carcinoma, and 3 had in situ melanoma (nasal tip and cheek). The 4 patients had lentigo maligna at the time of inclusion in the study.

A total of 56% of patients had a family history of cancer, and some patients had several affected relatives. Of these, the most frequent were lung cancer (20.93%) (9/54), gastric and uterine cancer (16.67%) (7/54), breast cancer (10%), cervical cancer (23.33%), prostate cancer (13.33%), and other types of cancer (10%).

A total of 46.29% (25/54) of the melanoma studied were invasive. For Breslow thickness, 20% (5/25) presented Breslow I (>0.76 mm), 20% (5/25) presented Breslow II (0.77-1.55 mm), 20% (5/25) presented Breslow III (1.56-4 mm), and 40% (10/25) presented a thickness greater than 4 mm. Regarding the Clark level, 31.82% (7/22) were Clark level II, 13.64% (3/22) were Clark level III, 45.45% (10/22) were Clark level IV, and 9.09 (2/22) were Clark level V.

Of the invasive melanomas, 36% (9/25) were histologic subtype lentigo maligna/lentigo maligna melanoma, 32% were nodular melanoma, 20% were ALM, and 12% were superficial spreading melanoma. A total of 36% of the invasive melanomas were located in the head and neck, 32% in the lower limbs, 20% in the trunk, and 12% in the arms. Additionally, 40% of melanomas showed histological ulceration.

### 3.2. Mutation Analysis

**Figure 1:**
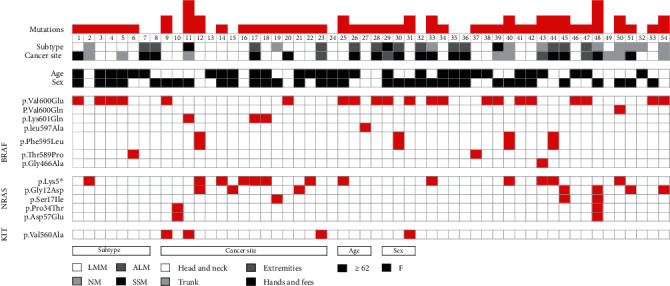
Mutations in the BRAF, NRAS, and C-KIT genes identified in 54 patients with cutaneous melanoma.

The presence of the V600E mutation in the BRAF gene and the mutations p.G12D, p.G12T, p.G13D, and p.Q61K in the NRAS gene were analyzed using specific probes. Mutations were verified by Sanger sequencing method for all exons analyzed. There was concordance between the mutations analyzed by qPCR and sequencing. Additionally, complete sequencing of the exons of these 2 genes made it possible to find other mutations with less recurrence. A total of 61% (32/54) of the patients had mutations in the BRAF gene. In these patients, a total of 33 mutations were found in exons 11 and 15 of this gene. Ninety-seven percent of the mutations were located in exon 15, and only 1 of the 33 mutations was detected in exon 11 (p.Gly466Ala). The most frequent mutation in the BRAF gene was p.Val600Glu, 63.6% (21/33). This mutation was present in 38.8% of the patients (Figure 1).

The majority of the samples positive for mutations in the BRAF gene had the following subtypes: LM/LMM (*n* = 19; 59.37%), ALM (*n* = 6; 18.75%), NM (*n* = 5; 15.62%), and SSM (*n* = 3; 9.37%) (Table 1). Of the melanomas with BRAF gene mutations, 15% (5/32) had a thickness less than 1 mm, 12.5% (4/32) a thickness between 1 and 2 mm, inclusive, 9.3% (3/32) a thickness greater than 2 mm up to 4 mm, and 21.8% (7/32) a thickness greater than 4 mm. Forty percent (13/32) had no thickness reported. Forty-seven percent (9/19) of the LM with mutations in the BRAF gene were invasive. Sixty-six percent of these (6/9) had the BRAF V600E mutation, and the other 33% (3/9) each had the p.Lys601Gln, p.Phe595 Leu, or p.leu597Ala mutation. Fifty percent of LM/LLM, 66% of SSM, 23% of ALM, and 20% of NM had mutation V600E (c.1799T > A). Thirty-eight percent of ALMs, 33% of SSMs, and 10% of LM/LMMs had mutations in the BRAF gene other than the p.Val600Glu mutation.

A total of 38.88% (21/54) of the patients showed mutations in the NRAS gene. In the 21 patients positive for variants in this gene, 5 variants were found between exons 2 and 3 (p.Lys5∗, p.Gly12Asp, p.Ser17Ile, p.Pro34Thr, and p.Asp57Glu). A total of 27 mutations in NRAS were detected. Ninety-two percent of these mutations were in exon 2. The recurrent variant was p.Lys5∗ (*n* = 13; 52%), followed by the p.Gly12Asp variant (*n* = 7; 28%); the remaining mutations were the p.Ser17Ile and p.Pro34Thr variants (12% and 8%, respectively). In exon 3, 8% of the mutations were presented (p.Asp57Glu variant). In the C-KIT gene, the variant p.Val560Ala (exon 11) was found in 7.40% (4/54) of the analyzed samples. Of the four patients with C-KIT gene mutations, two had LM/LMM, 1 had SSM, and 1 had ALM.

Eleven analyzed samples showed mutations in both the NRAS gene and the BRAF gene. Of these, 82% had the heterozygous mutation p.Lys5∗ in the NRAS gene. Eighteen percent (2/11) presented the BRAF V600 mutation, and 72% (8/11) presented mutations in another region of exon 15. Twelve percent (1/11) of these mutations were found in exon 11. (Figure 1).

## 4. Discussion

The patient sample analyzed in this study lives in a mountainous area of the equatorial region, where the population is exposed to high solar radiation indices (index > 10) [22]. However, in this region, the melanoma incidence is low compared to other areas of the world [23–25]. Therefore, an understanding of the mutation load in this population is necessary to relate the genotype and the already described differences in the frequency of histological subtypes of melanoma with other geographic areas of the world.

In this study, the average age of the patients was 62 years; these data are similar to those reported in the United States literature, where a peak incidence occurs between 55 and 64 years of age [26].

Of the total sample, 63% were women, and female predominance was maintained in all melanoma subtypes, being more marked in LM and ALM (61 and 62%, respectively). This differs slightly from that reported in the literature, where there is usually a higher incidence in men. However, this incidence can vary according to age; in this sense, it has been described that the incidence rate of MM is higher in women than in men up to 40 years, and then it begins to predominate in men, becoming 3 times more frequent in males at 75 years of age [27].

There is a clinical classification of melanoma that usually correlates with the histological classification. Historically, SSM has been the most frequent subtype of MM, and approximately 70% of all MMs are SSMs. This type of MM has been associated with acute intermittent exposure to the sun; its most frequent location is the back followed by the legs, in both men and women [27].

Unlike what has been described in the literature, in this sample of patients, the most common subtype of MM was LM/LMM 52% (28/54). This result is directly related to the most frequent tumor location (48% in the head and neck). Unlike our data, LM/LMM is described in the literature as one of the least common MMs, accounting for only 4 to 15% of MMs. Unlike NM and SSM, LM is correlated with long-term solar exposure [27].

ALM is a rare melanoma in Caucasians (5%); however, it has been described as the most common melanoma in Asian, Hispanic, and African patients [27]. In Colombia, the characterization of patients with cutaneous melanoma seen in the largest cancer reference center in Colombia found that ALM was the most frequent (44% of cases), followed by LM (24%) and SSM (14%) [28]. Although the present study is not an incidence study, it is worth noting that ALM was the second most frequent subtype (24%) followed by NM (19%).

In this study, the gene with the highest mutation frequency was BRAF (61%), followed by NRAS (37%), and C-KIT, which had an even lower frequency (7%). The V600E mutation in the BRAF gene was present in 38.8% of the patients analyzed. These data, in general, agree with those reported in the literature, where it has been described that mutations in 50% of primary melanomas present mutations in the BRAF gene, most originating from codon 600 in exon 15 (V600E) [29]; however, they differ in that the V600E mutation presents a lower frequency (38.8%) compared to that reported in other analyses.

BRAF V600 mutation has been found in 48-69% of patients with melanoma [30]. This prevalence is higher in Caucasian populations than in Asian populations [31]. In Latin America, there are few data, and the variation is large. For example, in Uruguay, a frequency of 78% has been reported, while in Córdoba and Argentina, the reported frequency is 16% [32, 33]. The frequency can also vary within the same country [13]. These differences reflect the racial and genetic ancestry variability found in Latin America, where the current population is a mixture of indigenous peoples, Africans, and Europeans. Likewise, this variation is not homogeneous; for example, in the south of the continent (Argentina, Chile, and Uruguay), the European component is substantial, but in Peru, Ecuador, and Bolivia, an indigenous component predominates. The origin of the patients analyzed in this sample is the Andean region of Colombia, where a genetic mixture of natives (34%), Africans (7%), and Europeans (57%) has been described [34]; this could explain in part the difference in the mutation frequency compared with other European or American regions.

The low frequency of BRAF V600 mutation found in our population (38.8%) also differs from that reported in the study by Carranza et al. [21], in which BRAF mutations were found in only 24% of cases; this result could be explained by their inclusion of cutaneous melanomas and uveal and mucosal melanomas. BRAF mutations are more frequent in SSM and in melanomas located on the trunk; in fact, in our study, although we only had 3 cases with SSM, all had BRAF mutations [31]. However, in our data, 59.37% of all the BRAF mutations found corresponded to patients with LM/LMM (19/32), which contrasts with that reported in the literature around the world [31] and with Latin American studies [13, 32], in which BRAF mutations occur in only 12% and 17% of LM/LMM cases, respectively. Related to the above, we found that 50% of all detected BRAF mutations corresponded to melanomas located on the scalp or face and 25% to melanomas located on the hands and feet. The progression from a premalignant lesion to melanoma is accompanied by an increase in the number of mutations in the MAPK pathway [35]; in this study, 76% of invasive melanomas presented mutations in the BRAF gene, and 28% (7/25) presented mutations in both the BRAF gene and the NRAS gene.

The BRAF V600 mutation has been associated not only with SSM but also with intermittent sun exposure [36]. Two meta-analyses published in 2011 and 2015 found that those patients with melanoma present a double risk of presenting BRAF mutations compared to patients with chronic sun exposure [10, 31]. However, the relationship between BRAF mutations and sun exposure is not clear [37]. Curtin et al. [38] describe a mutation frequency of 59% in MM in patients without chronic sun damage and a frequency of 11-23% in mucosae, soles, palms, and subungual MM. Therefore, these studies suggest that the greater is the sun exposure, the higher is the BRAF V600 mutation frequency [38]. In the study by Wu et al. [37], which evaluated the association between BRAF and NRAS mutations with phenotypic characteristics and a history of sun exposure in the NHS study (nurse health study) cohort in the United States, no clear association was found between phenotypic characteristics and mutations. Except for the association between the BRAF V600E mutation and living in a state with a UV index > 7 for 30 years (OR 5.54, 95% CI 1.19-25.8), there was no association between place of residence and NRAS mutations [37].

As reported in the literature, in this study, the most frequent BRAF mutation was BRAF V600 (38.8%, 21/54) [30]. This mutation was more prevalent in LM (67%, 14/21) followed by ALM (14%, 3/21). Thirty-seven percent of the detected BRAF mutations were different from V600. In contrast, 24% of melanomas positive for BRAF mutations were the histological subtype ALM, which is not related to sun exposure. Accordingly, the results suggest that location is correlated with a genetic profile that goes beyond histological classification.

The study by Wu et al. [37] in 2014 found lower survival of patients with BRAF mutations: the mean survival time for patients with MM and the BRAF V600E mutation was 100 months, compared to 128 months for those with the NRAS Q61R mutation for 159 months for patients without mutations. Although the association between BRAF mutations and tumor invasion (Breslow's thickness and Clark levels) was explored, no correlation was found. Conversely, Lee et al. [10] found that BRAF mutations were more frequent in cases of melanoma with a thickness less than 1 mm (OR 1.7), but in the meta-analysis by Kim et al. [31], there was no association between BRAF mutation and tumor thickness. In our study, no trend or possible association between BRAF mutation and tumor thickness was observed [10, 31].

The RAS-RAF-MEK-ERK pathway is the key for a variety of functions, including cell proliferation and cell survival [39]. Alterations in this signaling pathway have been described as early events in the progression of melanoma [40]. The genes with the most frequent mutations in melanoma are BRAF and NRAS. Mutations in the BRAF gene can be grouped into 2 regions: the glycine-rich region and the flanking region of the kinase activation segment [41]. The kinase domain (KD) of BRAF includes amino acids 457 to 717 [42].

In this study, the V600E recurrent genetic variant in the BRAF gene was found in a high percentage of LM/LMM samples, followed by ALM samples. The BRAF V600E mutation is considered a class I mutation due to the 500- and 700-fold increase in BRAF kinase activity compared to wild-type BRAF. This variant maintains the MAPK signaling pathway [42], which relates it to tumor biology.

A proportion of cutaneous melanomas show remnants of a nevus at the histological level [43]. This suggests that a possible route for the development of melanoma is the route likely to develop a nevus and that a high number of nevi is a risk factor for the development of melanoma [44]. There are reports that differ markedly in the number of melanomas associated with nevi; some figures estimate that this association occurs in up to 58% of cutaneous melanomas [45]. In the analyzed population, 88% of patients had a low number of nevi (<50); additionally, the incidence of superficial spreading melanoma is lower in Caucasians [46]. This suggests that a high number of nevi is not a risk factor for melanoma in general but is a risk factor for the development of superficial spreading melanoma located on the trunk. The BRAF V600 mutation has been associated with different histomorphological characteristics of nevi [47] and with the association between the total number of nevi and superficial spreading melanoma. However, 61% of the melanomas analyzed had a BRAF mutation, and as mentioned previously, the majority corresponded to the LM histological subtype and ALM. This finding is interesting because the BRAF mutation in the analyzed population can be interpreted as a risk factor for the development of melanoma independent of the total number of nevi and the histological subtype of melanoma.

In addition to the V600E variant, in this study, the presence of other variants was found throughout exons 11 and 15. Six different variants were found between amino acids 466 and 602 of the protein. The pGly466Ala variant was found in a patient with NM. This variant is located at the N-terminal end of KD in the glycine-rich loop-P, which is important for stabilizing adenosine triphosphate (ATP) binding and keeping the BRAF protein inactive. This variant is associated with an average increase in ERK activation [41]. The other variants, p.Thr589Pro, p.Phe595Leu, p.Leu597Ala, p.Lys601Gln, and p.Val600Gln, are in the kinase activation domain of the protein and are classified as class II, where kinase activity is intermediate to high [42]. We did not find a relationship between the presence of these mutations and the histological subtype.

Of the 54 melanoma samples analyzed, 38.8% had NRAS mutations. This frequency is higher than that reported in the literature, in which a frequency of 15-20% is reported for cutaneous melanoma [48]. Mutations in NRAS predominated in subtypes NM (43%, 9/21) and LM/LMM (38%, 8/21). Finding a high frequency of LM is consistent with what has been described in the literature, i.e., that NRAS mutations are associated with tumors in areas with continuous sun exposure [49, 50]. However, if we considered subtypes, the frequency was higher in NM; of the 10 patients with NM, 9 (90%) had NRAS mutations.

This suggests that although both proteins (B-Raf and N-Ras) activate the MEK-ERK pathway, the outcome is different. The mutations most frequently studied in NRAS are Q12 and Q61; however, in this study, the variant p.Lys5∗ (heterozygous) was present in 24% of samples, followed by the variant p.Gly12Asp in 13% and p.Ser17Ile in 5.5%. These findings confirm that mutations in the NRAS gene in the study population are the second most frequent, as has been reported in other studies.

Of the 100% of patients with mutations in the NRAS gene, 23% had a Breslow's thickness greater than 4 mm (5/21). The relationship between NRAS mutations and worse prognosis is inconclusive; some authors have associated rapid growth and lower survival with NRAS mutations, while other authors find a better prognosis in patients with MM and NRAS mutations [17, 51].

Of the patients who we found had BRAF mutations, 34.37% (11/32) also had NRAS mutations. This is contrary to what has been reported because these mutations are exclusive and rarely overlap [37].

In contrast, C-KIT mutations in this study occurred in less than 10% of samples, despite the frequency of ALM found in 24%. Additionally, the mutation found (p.Val560Ala) was only detected in one case of ALM. This differs from the concept that C-KIT mutations are more frequently found in mucosal and acral MM than in melanomas in individuals who live in areas with intermittent exposure to the sun. However, more recent studies mention a low frequency of such mutations [52].

An important finding in the samples analyzed is the rare genotypes, such as the presence of NRAS and BRAF genes mutations in the same sample. However, it has been reported that NRAS and BRAF gene mutations are mutually exclusive [53]. Ellerhorst et al. [49] reported 3 patients with the presence of samples with double mutations. This is in agreement with the results found here, in which 11 analyzed samples presented this characteristic.

The findings of this study, such as the high frequency of BRAF V600 mutations in both LM/LMM and ALM and the heterogeneity of the mutations in the 2 genes (BRAF and NRAS), suggest that mutations are early events in the development of melanoma and that other factors may have a greater impact on the aggressiveness of cutaneous melanoma.

## 5. Conclusions

In conclusion, the recurrent variant BRAFV600E represented 63.63% of all mutations in this gene in the samples analyzed, indicating that it is important to evaluate the complete exon 15. The recurrent variant (p.Lys5∗) found in the NRAS gene in the analyzed samples was different from that reported in other parts of the world. Furthermore, it was confirmed that mutations in the BRAF and NRAS genes are not always mutually exclusive. The frequency of the BRAF, NRAS, and C-KIT mutations is different in different geographic regions.

## Figures and Tables

**Table 1:** Mutation analysis by gene and patient clinicopathologic characteristics.

	Mutation **BRAF** = 32	WT **BRAF** = 22	Mutation **NRAS** = 21	WT **NRAS** = 33	Mutation **C** − **KIT** = 4	WT **C** − **KIT** = 50
Mean age	61.27 (27-81)	63.73 (41-86)	61.38 (30-86)	62.63 (27-85)	56.5 (44-74)	62.2 (27-86)
Sex						
Man	13	7	7	13	0	20
Woman	22	12	14	20	4	30
Anatomical site						
Trunk	6	2	5	3	1	7
Extremities	5	2	1	3	1	3
Feet and hands	8	5	6	10	0	16
Head and neck	16	10	9	17	2	24
Melanoma subtype						
Lentigo maligna melanoma	19	9	8	20	2	26
Superficial spreading melanoma	3	0	1	2	1	2
Acral lentiginous melanoma	6	7	3	10	1	12
Nodular melanoma	5	5	9	1	0	10
Phototype						
I	6	2	1	7	2	6
II	25	16	17	24	2	39
III	3	1	3	1	0	4
IV	1	0	0	1	0	1
Clark						
II	6	1	4	3	2	5
III	2	1	1	2	0	3
IV	8	2	4	6	0	10
V	1	1	2	0	0	2
Breslow						
0.1 mm to 1 mm	5	0	1	4	1	4
>1 mm to 2 mm	4	1	3	2	1	4
>2 mm to 4 mm	3	2	2	3	0	5
>4 mm	7	3	5	5	0	10
Ulceration						
Present	8	2	6	4	0	10
Absent	11	4	6	9	2	13
Nevus						
<50	31	17	18	30	2	46
50-100	2	2	3	1	1	3
>100	2	0	0	2	1	1

## Data Availability

The files related to informed consent, clinical data, and molecular biology results; data used to support the findings of this study are restricted by ethics committee of the hospital CENTRO DERMATOLOGICO FEDERICO LLERAS ACOSTA in order to protect patient privacy. However, the data are available from de file of code 4000.16.6AC for researchers who meet the criteria for access to confidential data. These files can be requested at the following email from the ethics committee: Comitedeeticaeninvestigacion@dermatologia.gov.co.
